# OIP5-AS1 contributes to the development in endometrial carcinoma cells by targeting miR-152-3p to up-regulate SLC7A5

**DOI:** 10.1186/s12935-021-02061-0

**Published:** 2021-08-21

**Authors:** Minglin Liang, Hongbo Wang, Cong Liu, Tao Lei, Jie Min

**Affiliations:** Department of Obstetrics and Gynecology, Union Hospital, Tongji Medical College, Huazhong Univercity of Science and Technology, No. 2177 Liberation Avenue, Wuhan, 430022 China

**Keywords:** OIP5-AS1, miR-152-3p, SLC7A5, Endometrial carcinoma

## Abstract

**Background:**

Endometrial carcinoma (EC) is one common gynecological tumor, threatening physical and psychological health of females. Huge amount of essays indicated that long non-coding RNAs (lncRNAs) were widely reported to serve as a crucial regulator in the biological movements among multiple carcinomas, including EC.

**Methods:**

RT-qPCR was implemented to detect the expression of target genes. Loss/gain-of-function experiments certified the impacts of OIP5-AS1 and miR-152-3p on EC cell progression.

**Results:**

Data of this research suggested that powerful expression of OIP5-AS1 was discovered in EC cell lines. Loss/gain-of-function assays inferred that OIP5-AS1 promoted proliferative, migratory and invasive abilities, and Epithelial-Mesenchymal Transition (EMT). In addition, we identified miR-152-3p expression was negatively modulated by OIP5-AS1. OIP5-AS1 accelerated the development of EC cells via downregulating miR-152-3p expression. SLC7A5 was selected out as a downstream target of miR-152-3p. The competing relationship between OIP5-AS1 and SLC7A5 was corroborated by luciferase reporter assay. Eventually, the results of rescue assays indicated that SLC7A5 overexpression could restore the impacts of OIP5-AS1 ablation on the progression of EC cells.

**Conclusion:**

Our research confirmed that OIP5-AS1 propeled the development of EC cells through targeting miR-152-3p/SLC7A5. OIP5-AS1 could be utilized as a target for EC treatment.

**Supplementary Information:**

The online version contains supplementary material available at 10.1186/s12935-021-02061-0.

## Background

Endometrial carcinoma (EC) is considered as one of the most widespread gynecological malignancies in European Union, with more than 73,000 cases diagnosed in 2020[1]. It is first and foremost to choose surgical operation as prioritized therapeutic methods for EC treatment. With the consolidation of adjuvant chemotherapy and radiotherapy, the survival rate is improved and progression is delayed in EC [2, 3]. In the late decades, although great progress has been made in therapies for EC, the prognosis of EC patients still remains poor [4]. The recurrence rate is high due to the metastasis in the end-stage of EC. Therefore, it is vital to probe into the pathology mechanism of EC and ascertain a remedy target to improve the treatment.

LncRNAs are a cluster of transcripts with over 200 bases in length and without the ability to translate proteins [5]. Emerging evidence has attested the importance of lncRNAs in the process of various cancers. For example, LINC01170 accelerated the growth of EC via activation of AKT pathway [6]. PVT1 acted as an oncogene in ovarian cancer through enhancing SOX2 [7]. The regulatory role of OIP5-AS1 was studied in osteosarcoma [8], lung cancer [9] and colorectal cancer [10]. Nevertheless, the link between OIP5-AS1 and EC still remains to be explored.

MicroRNAs (miRNAs) are defined as the RNAs featuring about 18–25 nucleotides in length and inability to code proteins, and they can modulate target gene expression through interacting with the 3’ untranslated region (UTR) of the mRNAs, which are their downstream targets [11]. Accumulating researches indicated that miRNAs were active regulators of the proliferation, migration and chemoresistance in numerous cancers. For instance, miR-21 acted as an oncogene in tongue squamous cell by promoting proliferation and invasion in vitro [12]. MiR-630 suppressed metastasis in esophageal squamous cell carcinoma [13]. MiR-135b accelerated proliferation of EC through targeting FOXO1 [14]. MiR-152-3p modulated progression of prostate cancer by inhibiting KLF4 synergistically with miR-148-3p. However, the detailed function of miR-152-3p has not been explained thoroughly in EC.

Our main aim was to probe into the mechanisms and functions of OIP5-AS1 in EC cells and the interaction between miR-152-3p and OIP5-AS1 in our study. Furthermore, the underlying targets of miR-152-5p would also be investigated.

## Materials and methods

### Cell lines and tissue samples

EC cell lines (Ishikawa and ECC), and the normal cell line (SHT290) were procured from the Cell Resource Center of Chinese Academy of Medical Sciences (Beijing, China), then cultivated in the Dulbecco’s modified Eagle’s medium (DMEM; Gibco, Grand Island, NY). Cell culture was implemented with 10% fetal bovine serum (FBS; Gibco) as medium supplement, in the 95% air/5% CO_2_ incubator at 37 °C.

### RNA extraction and real-time quantitative PCR (RT-qPCR)

The total cellular RNAs were severally extracted on the basis of the protocol of TRIzol reagent (Invitrogen, Carlsbad, CA). 3 μg of total RNAs were converted into the cDNA by applying the Reverse Transcription Kit (Toyobo, Osaka, Japan). Gene expression was quantified by ABI Step-One Plus Real-Time PCR System (Applied Biosystems, Foster City, CA) with the SYBR® Premix Ex Taq™ II (Takara). As for Data calculation, 2^−ΔΔCT^ method was used, with GAPDH and U6 as controls.

### Cell transfection

The short hairpin RNAs (shRNAs) against OIP5-AS1 were constructed by Genepharma (Shanghai, China) for gene silencing analysis, as well as its control, sh-NC. The pcDNA3.1/OIP5-AS1, pcDNA3.1/SLC7A5 and the control pcDNA3.1 vectors were commercially obtained from Ribobio (Guangzhou, China) to up-regulate target genes. Besides, the miR-152-3p mimics/inhibitor, as well as the corresponding negative control (NC mimics/inhibitor) were designed at Genepharma. Plasmids were transfected into EC cell for 48 h via Lipofectamine 2000 (Invitrogen).

### Colony formation

Clonogenic cell samples were cultivated as 500 cells per well into the 6-well plates for 14 days, followed by the treatment with the 0.5% crystal violet staining solution in 4% paraformaldehyde. Clones were determined and counted.

### Flow cytometry analysis of apoptosis

Flow cytometry analysis was performed for the examination of cell apoptosis. The cells were stained in Annexin V-labeled with 7AAD and PE solution (BD Biosciences, San Jose, CA) for a quarter of an hour in the dark. After being rinsed in the pre-chilled phosphate-buffered saline (PBS) and double-staining, cell samples were assayed by FACS cytometry (BD Biosciences) finally.

### Western blot

30–50 mg of the cellular total proteins were subjected to RIPA lysis buffer for isolation and then to 12% SDS-PAGE gel for separation. Subsequently, the separated protein samples were electro-blotted onto the PVDF membranes (Millipore, Billerica, MA), and then treated with the 5% skimmed milk solution for blocking. Samples were cultivated with the primary antibodies against the loading control GAPDH and Bcl-2, Bax, Cleaved caspase-3, Total caspase-3, as well as four EMT markers, slug, Vimentin, N-cadherin, E-cadherin (at 1: 2000 dilution, Abcam, Cambridge, MA) for a whole night. Followed by rinsing in the Tris-buffered saline (TBST), samples were incubated with the secondary antibodies (at 1: 5000; Abcam) for 2 h. Blots were finally detected by Immobilon® Western Horseradish peroxidase substrate (Millipore).

### Transwell assays

6 × 10^4^ cell samples in the 100 ml of medium free of FBS were placed into the upper part of the Transwell chamber (Corning Life Sciences, Corning, NY). 600 ml of conditioned medium was placed into the lower chamber. Followed by 24 h of incubation at 37 °C, migrating cell samples were fixed for 15 min and treated with 0.5% crystal violet staining for 30 min. The up‐right fluorescence microscope (200×) was applied for counting. Cell invasion was analyzed with Transwell chamber coated by the Matrigel membrane (BD Biosciences).

### FISH assay

Cell samples were first fixed for 15 min, then rinsed in PBS and air-dried. 40 nm of the FISH probe synthesized for OIP5-AS1 was procured from Ribobio and mixed with the cell samples in the hybridization buffer. As for the counterstaining of nucleus, Hoechst solution was adopted. The final images were captured using the fluorescence microscope.

### RNA pull down

The assay was conducted by employing the Pierce Magnetic RNA–Protein Pull-Down Kit (Thermo Fisher Scientific, Waltham, MA) in light of the user manual. The wild-type (WT) or mutated (Mut) miR-152-3p sequences with the OIP5-AS1 binding sites were constructed, followed by the biotinylation into Bio-miR-152-3p-WT/Mut probes. The cells, after being lysed, were subjected to the 1 h-incubation with the probes and streptavidin agarose magnetic beads. After RNA extraction and purification, RT-qPCR was finally conducted for determining OIP5-AS1 enrichment in pull-downs.

### Dual-luciferase reporter assays

OIP5-AS1-WT/Mut or SLC7A5-WT/Mut fragments covering the miR-152-3p interacting sequences were constructed and subjected to the subcloning into the pmirGLO Dual-Luciferase Expression Vector (Promega, Madison, WI). Afterwards, the reporter vectors were subjected to the co-transfection into EC cells along with the specific plasmids for 48 h. Dual-Luciferase Reporter Assay System (Promega) was employed for luciferase activity.

### RNA immunoprecipitation (RIP)

The assay was implemented on a basis of the instruction of EZ-Magna RIP RNA Binding Protein Immunoprecipitation Kit (Millipore). The cultured cell samples were lysed in the RIP lysis buffer, then collected and treated with magnetic beads bound to the antibodies of Ago2 or IgG (Millipore) in RIP buffer. Following RNA extraction, the purified RNAs were assayed by RT-qPCR.

### Statistical analysis

All assays were subjected to independent bio-repetition in triplicate. All the experimental results were represented as the mean ± standard deviation (SD). Data analysis was developed by Student's t-test and one-way or two-way analysis of variance (ANOVA) employing the Prism 5.0 (GraphPad Software Inc., La Jolla, CA). Significant level was set as the p-value less than 0.05.

## Results

### OIP5-AS1 is up-regulated in EC cells and boosts the development of EC cells

**Fig. 1:**
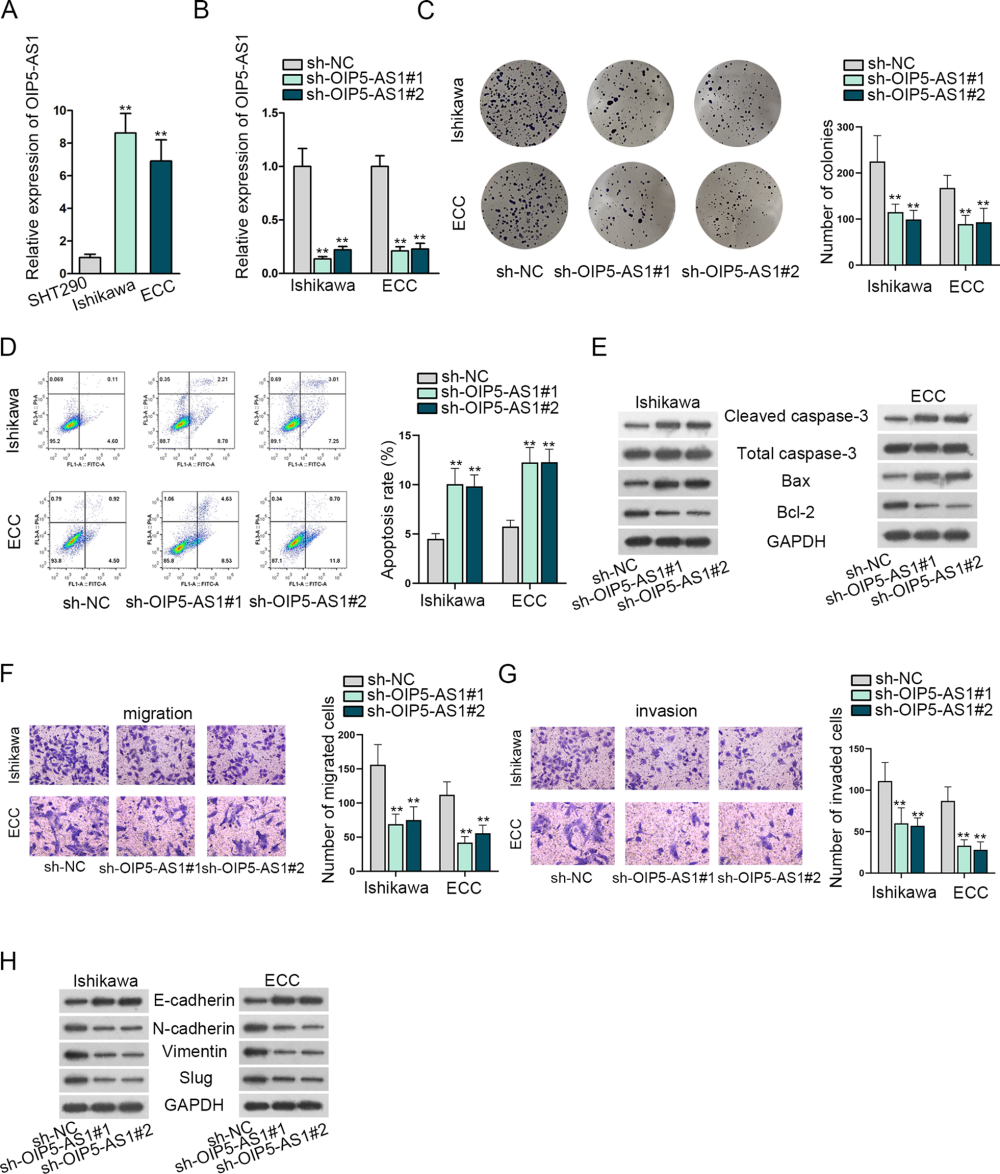
OIP5-AS1 isup-regulated in EC cells and boosted the development of EC. **A** OIP5-AS1 in Ishikawa and ECC cells by RT-qPCR. **B** Efficacy assessment of OIP5-AS1 silence through RT-qPCR in Ishikawa and ECC cells. **C** Cell proliferative ability evaluation in OIP5-AS1-ablated Ishikawa and ECC cells through the implementation of colony formation assays. **D** Cell apoptosis evaluation in transfected cells through flow cytometry. (E) Apoptosis-related proteins in transfected cells via western blot. **F**, **G** Cell migration and invasion assessment in transfected cells through transwell assays. (H) Estimate of associated proteins of EMT by western blot in transfected cells. **P < 0.01

At the beginning, we unveiled the potential role of OIP5-AS1 in EC. We observed from RT-qPCR a high expression of OIP5-AS1 in Ishikawa and ECC cells compared with SHT290 (Fig. 1A). Next, RT-qPCR testing was implemented for the detection of the knockdown efficacy of sh-OIP5-AS1#1/2 in Ishikawa and ECC cells (Fig. 1B). Furthermore, we performed loss-of-functional assays to confirm the influences of OIP5-AS1 on Ishikawa and ECC cells. Data from colony formation assays showcased that OIP5-AS1 silence decreased proliferation abilities in colony formation assays (Fig. 1C). On the contrary, OIP5-AS1 knockdown increased the apoptosis rate prominently (Fig. 1D). Western blot measured the associated proteins of apoptosis. According to the results, Cleaved caspase-3 and Bax proteins were elevated while Bcl-2 protein was decreased by OIP5-AS1 silencing (Fig. 1E). Results of transwell exhibited that the abilities to migrate and invade were weakened by OIP5-AS1 down-regulation (Fig. 1F, G). The EMT markers were measured via the implementation of western blot. Consequences displayed that E-cadherin was up-regulated while N-cadherin, vimentin, as well as Slug were lessened by OIP5-AS1 silence (Fig. 1H). To further verify the effect of OIP5-AS1, we subsequently conducted gain-of-functional assays in Ishikawa and ECC cells, with detecting the overexpression efficiency of pcDNA3.1/OIP5-AS1 first (Additional file 1: Fig. S1A). The results of functional assays and western blot showed that overexpressed OIP5-AS1 enhanced the development, and suppressed apoptosis of Ishikawa and ECC cells (Additional file 1: Fig. S1B–F). Taken together, OIP5-AS1 is high-expressed in EC cells, and OIP5-AS1 promotes the process of EC cells.

### OIP5-AS1 negatively regulates miR-152-3p in EC cells

**Fig. 2:**
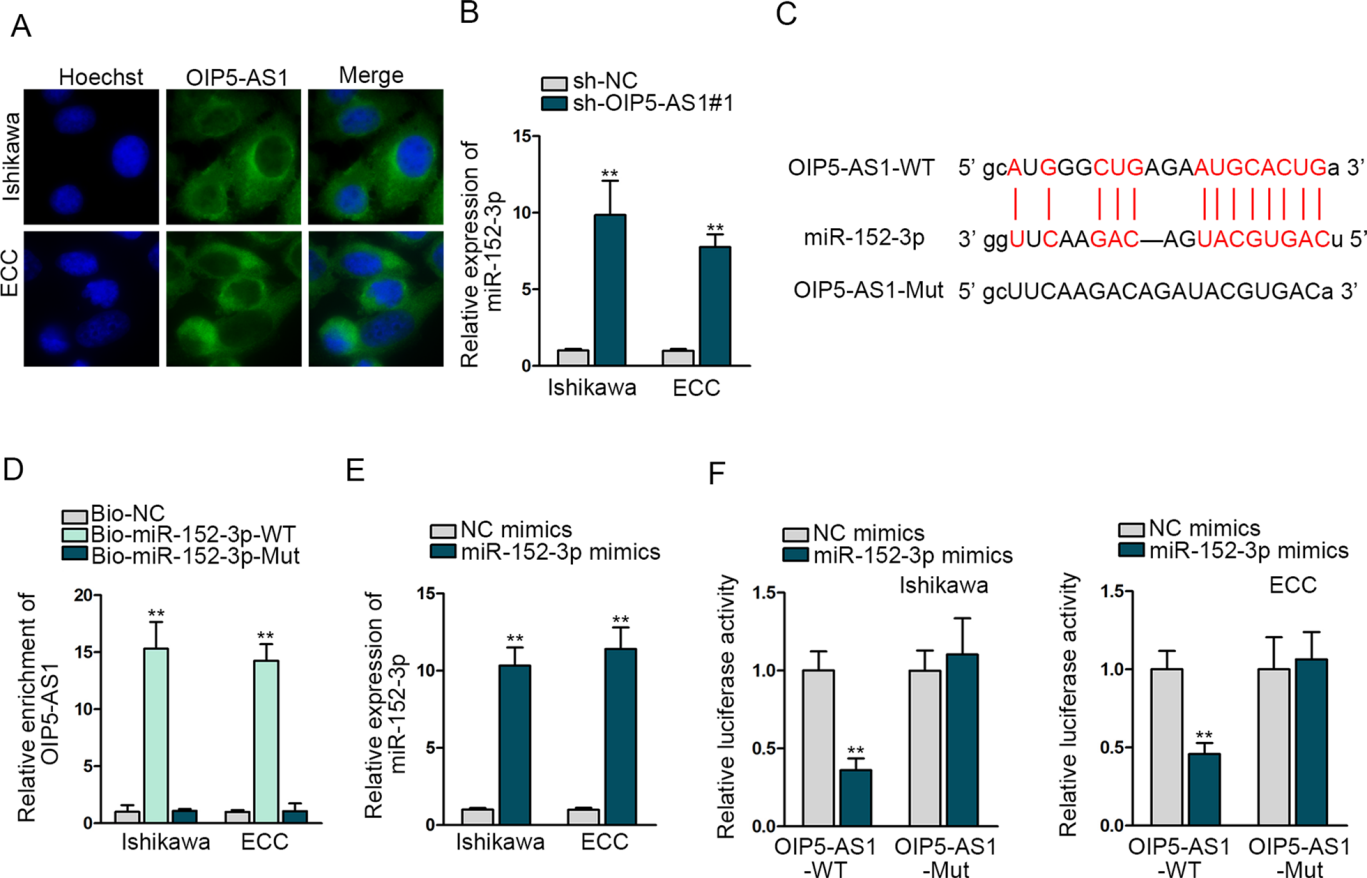
OIP5-AS1 negatively regulates miR-152-3p in EC cells. **A** The location of OIP5-AS1 in EC cells by FISH. **B** The detection of miR-152-3p expression after the decrease of OIP5-AS1. **C** Bioinformatics presentation of OIP5-AS1 and miR-152-3p binding sites. **D** RNA pull-down verified the correlation between OIP5-AS1 and miR-152-3p. **E** MiR-152-3p up-regulation efficiency examined in Ishikawa and ECC cells. **F** The verification of OIP5-AS1 and miR-152-3p interaction by luciferase reporter assay. **P < 0.01

For further exploration of OIP5-AS1 in EC cells, we carried out FISH assay and the outcomes disclosed that the main distribution of OIP5-AS1 was in cytoplasm in EC cells (Fig. 2A). Therefore, we raised a hypothesis that OIP5-AS1 regulates EC cells through ceRNA mode. Then, we searched http://starBase.sysu.edu.cn/ for potential miRNAs containing potential binding sites with OIP5-AS1. Among all the candidates, miR-152-3p was linked to the endometrial receptivity status [15]. Therefore, we selected miR-152-3p for the following-up experiments. After the transfection with sh-NC and sh-OIP5-AS1#1, RT-qPCR measured the expression of miR-152-3p in Ishikawa and ECC cells. Data revealed that miR-152-3p expression was escalated significantly (Fig. 2B), showing that OIP5-AS1 expression had a negative correlation with miR-152-3p expression. The binding sequences between miR-152-3p and OIP5-AS1 were presented in Fig. 2C. Subsequently, RNA pull down showed that biotinylated miR-152-3p-WT, instead of miR-152-3p-Mut, could pull down OIP5-AS1 into sediment, proving their interaction (Fig. 2D). Consequences of RT-qPCR delineated that miR-152-3p expression was elevated in response to miR-152-3p mimics (Fig. 2E). Data of luciferase reporter assays manifested that miR-152-3p up-regulation could dwindle the luciferase activity of plasmid with OIP5-AS1-WT instead of OIP5-AS1-Mut, further verifying the combination (Fig. 2F). In a word, OIP5-AS1 negatively regulates miR-152-3p in EC cells.

### Up-regulation of miR-152-3p suppresses the process of EC cells

**Fig. 3:**
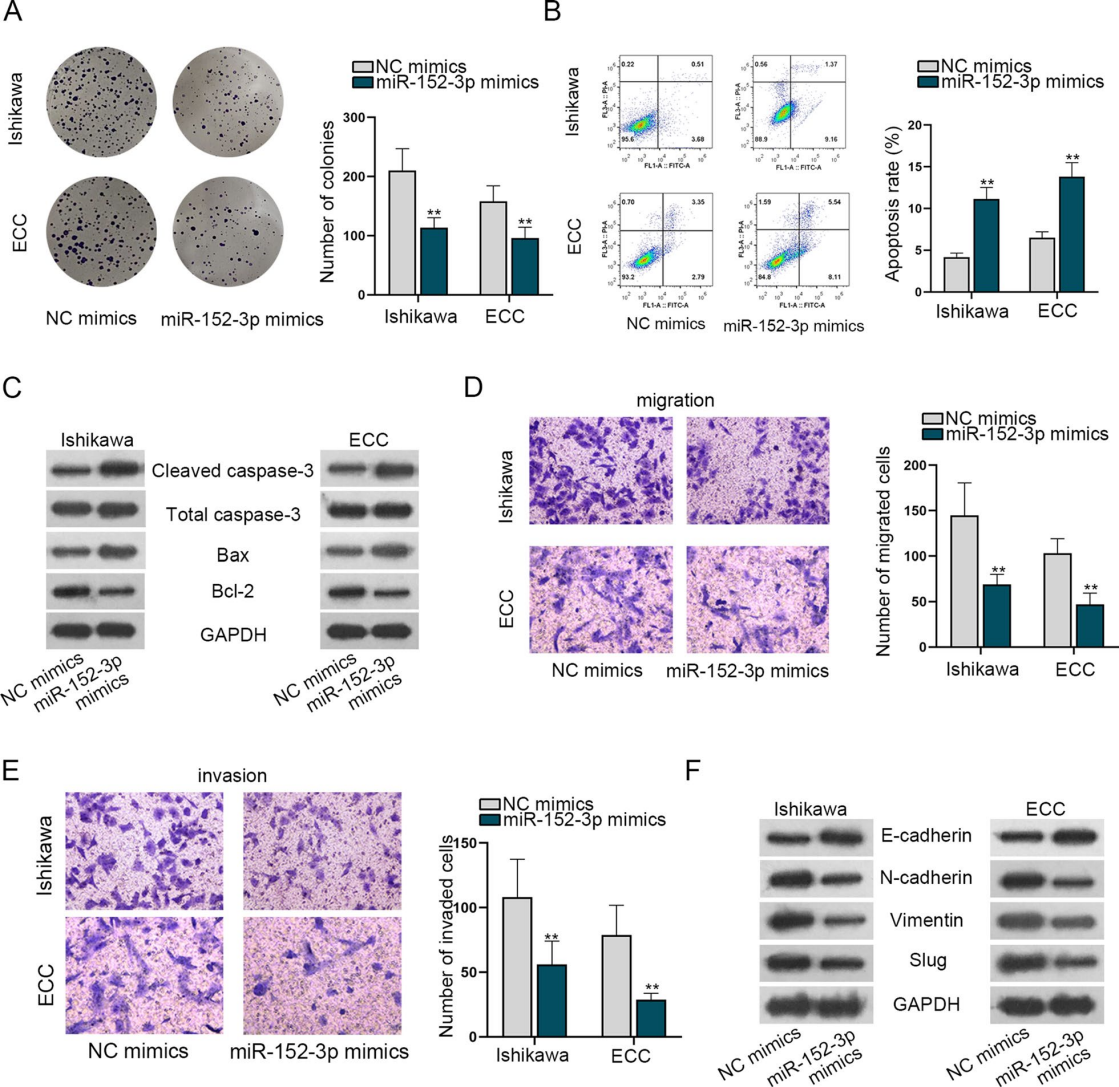
Up-regulation of miR-152-3p suppresses the process of EC cells. **A** Evaluation of cell proliferation ability in Ishikawa and ECC cells after miR-152-3p enhancement by colony formation assays. **B** Cell apoptosis validated in transfected cells by flow cytometry. **C** Associated proteins of apoptosis in transfected cells by western blot. **D**, **E** Transwell assays were set up to assess migration and invasion. **F** Related proteins of EMT were appraised by western blot. **P < 0.01

We designed functional assays to attest the biological functions of miR-152-3p in EC cells. Results of colony formation disclosed that miR-152-3p overexpression repressed the proliferation of EC cells (Fig. 3A). Moreover, miR-152-3p up-regulation lifted the apoptosis rate of EC cells (Fig. 3B). The proteins of apoptosis were changed in answer to miR-152-3p up-regulation as well (Fig. 3C). The migratory and invasive capacities were attenuated in transwell assays due to miR-152-3p overexpression (Fig. 3D, E). EMT of EC cells was suppressed after the overexpression of miR-152-3p as evidenced by western blot (Fig. 3F). In conclusion, miR-152-3p up-regulation hinders the process of EC cells.

### OIP5-AS1 contributes to the growth of EC cells via down-regulating miR-152-3p expression

**Fig. 4:**
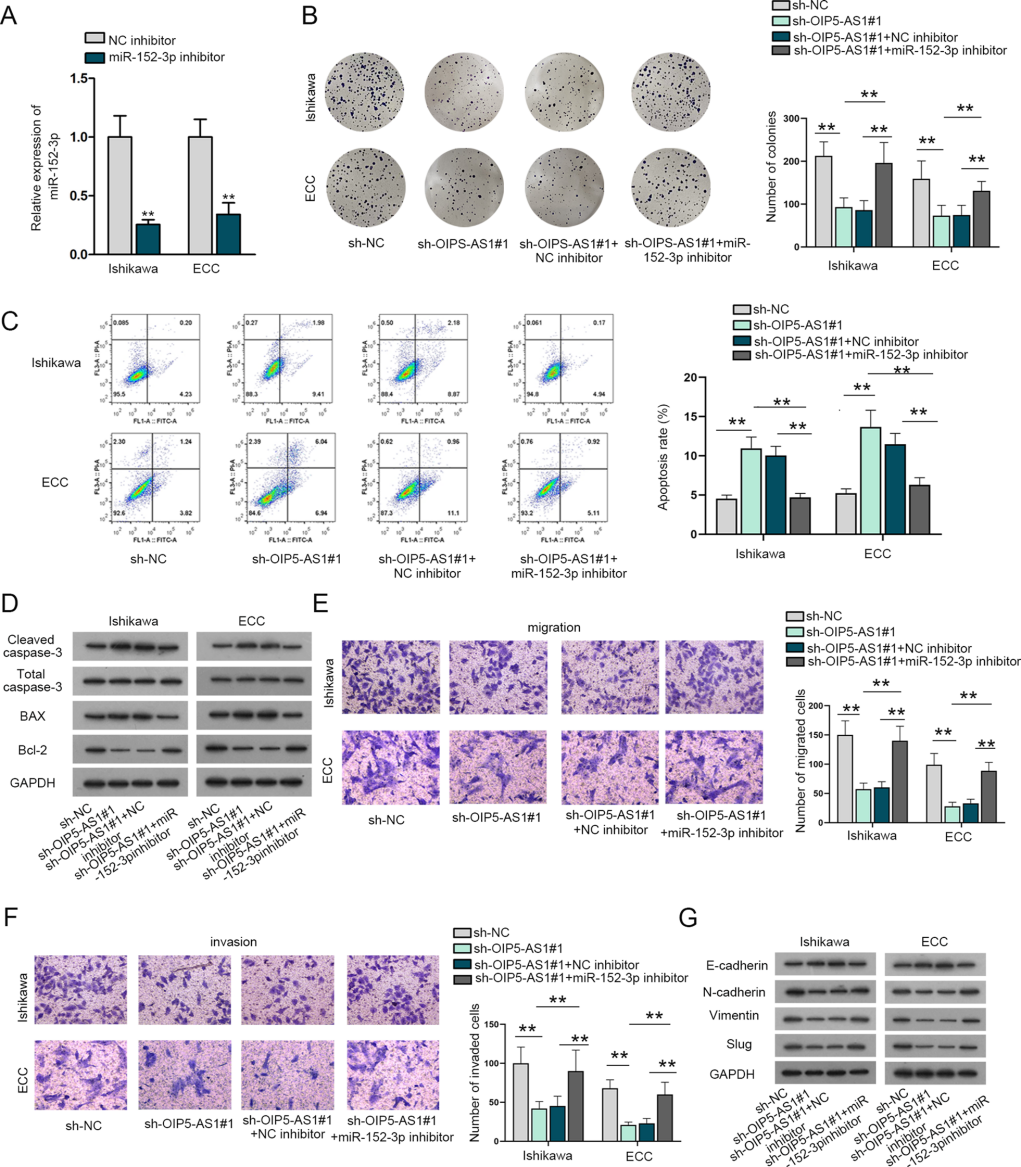
OIP5-AS1 contributes to the growth of EC cells via down-regulating miR-152-3p expression. **A** MiR-152-3p down-regulation efficiency examined in Ishikawa and ECC cells. **B** EC cells were treated with sh-NC, sh-OIP5-AS1#1, sh-OIP5-AS1#1 + NC inhibitor, sh-OIP5-AS1#1 + miR-152-3p inhibitor and colony formation assays evaluated the proliferation of transfected cells. **C** Flow cytometry analysis assessed cell apoptosis rate in transfected cells. **D** Western blot evaluated apoptosis-related proteins in transfected cells. **E**, **F** Transwell assays tested migratory and invasive abilities of transfected cells. **G** Western blot measured proteins of EMT of transfected cells. **P < 0.01

To study how OIP5-AS1 regulated miR-152-3p to affect the EC progression, we performed the rescue assays. MiR-152-3p inhibitor was transfected into cells for efficiency analysis (Fig. 4A). Next, we conducted a series of rescue experiments. The declined proliferative capacities imposed by OIP5-AS1 knockdown were enhanced by miR-152-3p down-regulation (Fig. 4B). The increased apoptosis induced by OIP5-AS1 silence were offset via down-regulated miR-152-3p (Fig. 4C). The elevated cleaved caspase-3 and Bax proteins along with decreased Bcl-2 proteins, which were caused by OIP5-AS1 knockdown, were countervailed by miR-152-3p depletion (Fig. 4D). In terms of transwell assays, down-modulated OIP5-AS1 dropped the capacities of EC cells to migrate and invade, which could be restored by miR-152-3p inhibitor (Fig. 4E, F). The relevant proteins of EMT were changed due to OIP5-AS1 down-regulation. After miR-152-3p inhibitor was transfected into Ishikawa and ECC cells, this trend was reversed (Fig. 4G). In brief, OIP5-AS1 facilitates the growth of EC cells through decreasing miR-152-3p expression.

### SLC7A5 overexpression can rescue the influences of OIP5-AS1 down-regulation on EC cell progression

**Fig. 5:**
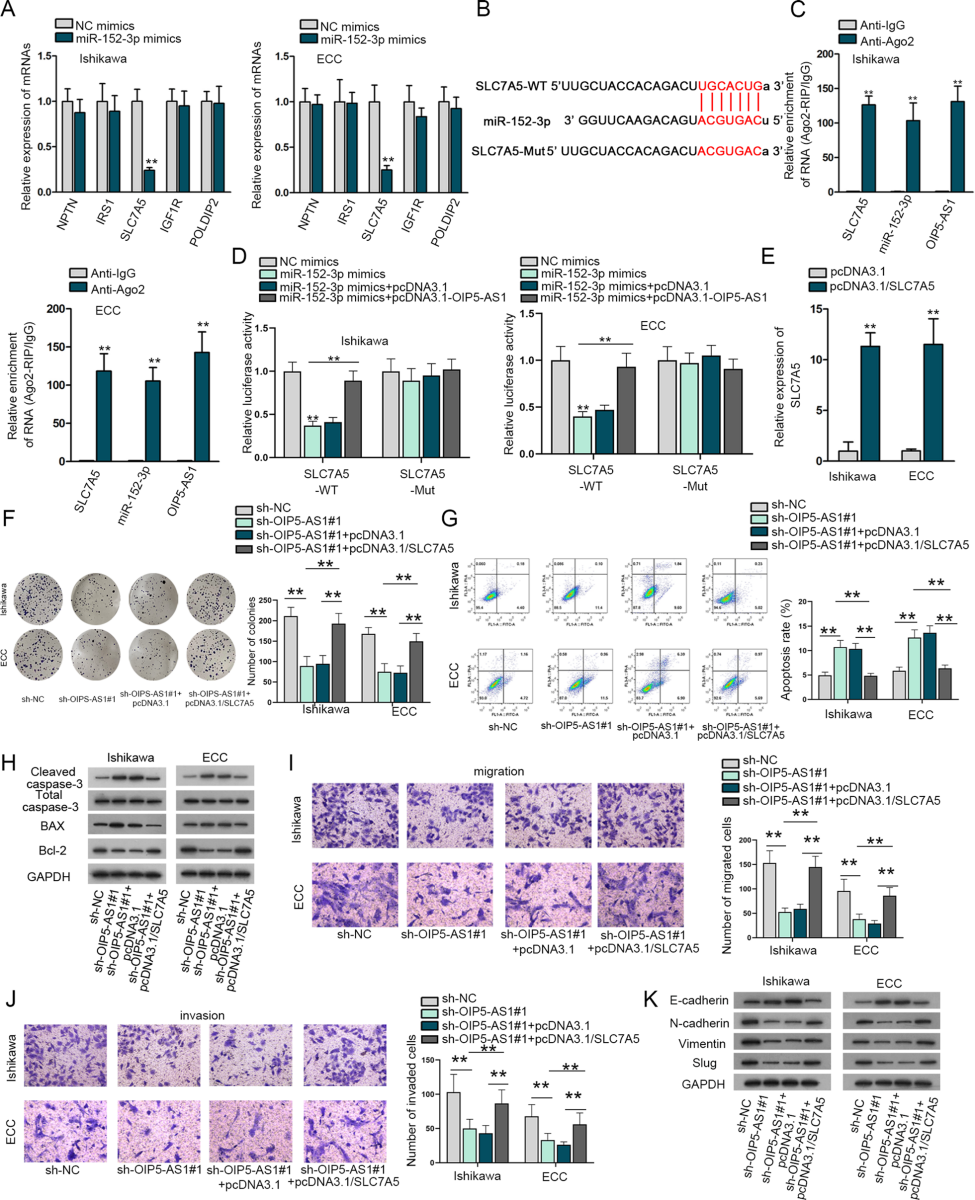
SLC7A5 overexpression can rescue the effects of OIP5-AS1 down-regulation on the development of EC cells. **A** These mRNAs expressions were detected by RT-qPCR in cells after the overexpression of miR-152-3p. **B** Bioinformatics presentation of miR-152-3p and SLC7A5 binding sites. **C** RIP assay testified OIP5-AS1, miR-152-3p and SLC7A5 coexisted in RNA induced silencing complexes (RISCs). **D** Luciferase activity of SLC7A5-WT/Mut in response to NC mimics or miR-152-3p mimics in EC cells as well as the addition of pcDNA3.1-OIP5-AS1 into cells. **E** Overexpressed SLC7A5 was validated in RT-qPCR. The influence of sh-OIP5-AS1#1 on EC cells proliferation (**F**), apoptosis (**G**, **H**), and migration as well as invasion (I-J) and EMT (K) was rescued by overexpression of SLC7A5. **P < 0.01

It is well recognized that the downstream targets-mRNAs could be translated into proteins to exert functions on the diseases. We searched starBase and found NPTN, IRS1, SLC7A5, IGF1R and POLDIP2 had latent binding sites with miR-152-3p. These mRNAs expressions were tested in miR-152-3p-overexpressed Ishikawa and ECC cells. Data showed that only SLC7A5 expression was lessened overtly while no changes could be seen in other mRNAs (Fig. 5A). The binding sites between miR-152-3p and SLC7A5 were exhibited in Fig. 5B. Results of RIP portrayed the coexistence of SLC7A5, OIP5-AS1 and miR-152-3p in RNA-induced silencing complexes (RISCs) (Fig. 5C). The luciferase activity of SLC7A5-WT group was undermined by miR-152-3p mimics, which was recovered by OIP5-AS1 overexpression. Considering the luciferase activity of SLC7A5-Mut group, no obvious changes were discovered (Fig. 5D). Additionally, we applied rescue assays to verify OIP5-AS1 modulated SLC7A5 to affect the progression of EC cells. Firstly, and the efficiency of pcDNA3.1/SLC7A5 was assessed in Ishikawa and ECC cells (Fig. 5E). The outcomes of rescue assays manifested that up-regulation of SLC7A5 could countervail the changes in proliferation (Fig. 5F), apoptosis (Fig. 5G, H), migration and invasion (Fig. 5I, J) as well as EMT of EC cells (Fig. 5K), which were induced through OIP5-AS1 knockdown. To sum up, OIP5-AS1 accelerates the growth of EC cells by up-regulating SLC7A5.

## Discussion

According to previous researches, lncRNAs are regarded as underlying biomarkers in human cancers and non-tumors. For example, lncRNA ZNF180-2 was considered as a prognostic biomarker among clear cell renal cell carcinoma [16]. LncRNA PANDAR promoted proliferation, cell cycle in thyroid cancer cell [17]. LncRNA-TSI inhibited renal fibrogenesis through modulating the TGF-β/Smad3 pathway negatively [18]. OIP5-AS1 was analyzed in lung adenocarcinoma [19]. Based on these precedent findings, the focus of this study is the function of OIP5-AS1 in EC. Intriguingly, OIP5-AS1 was observed being highly expressed in EC cells. Accordingly, OIP5-AS1 down-regulation suppressed proliferative, migratory and invasive capacities, and EMT while fostering apoptosis in EC progression.

Lately, accumulating attention was attracted to competing endogenous RNA (ceRNA) system. Supporters believed that lncRNAs could function as targets of miRNAs so that their downstream targets could be liberated to translate into proteins [20]. For instance, RSU1P2 boosted the cervical cancer growth via serving as a ceRNA against let-7a [21]. XIST was reported to exert oncogenic function in colorectal cancer via targeting miR-132-3p [22]. In this study, we testified the subcellular localization of OIP5-AS1 in EC cells. OIP5-AS1 was affirmed as a target of miR-152-3p. In addition, the downstream target SLC7A5 was selected out to be negatively modulated by miR-152-3p. The competing relationship between OIP5-AS1 and SLC7A5 was confirmed by luciferase reporter assays. More importantly, OIP5-AS1 could boost the progression of EC cells via up-regulating SLC7A5.

Increasing evidence demonstrated the crucial effects of miRNAs on the development and progression of human tumors. Prior researches have validated that miR-152 worked as a tumor inhibitor in breast cancer via modulating PIK3CA [23]. MiR-152-3p modulated hepatic glycogenesis through targeting PTEN [24]. Moreover, miR-152-3p down-regulation could rescue the effects of HOTAIR silence on the process of malignant melanoma [25]. In our study, it was found that miR-152-3p could be negatively regulated by OIP5-AS1. Moreover, miR-152-3p enhancement could restrict the growth of EC cells. Besides, miR-152-3p depletion could neutralize the influences imposed by OIP5-AS1 knockdown.

## Conclusions

In summary, the data of our study disclosed that OIP5-AS1 is able to foster the abilities of cell to proliferate, migrate and invade via targeting miR-152-3p to up-regulate SLC7A5 in EC cells, suggesting OIP5-AS1 might be a valuable target against EC.

## Supplementary Information

**Additional file 1: Figure S1.** (A) Efficacy assessment of OIP5-AS1 overexpression via RT-qPCR in Ishikawa and ECC cells. (B) Detection of proliferation capacity of Ishikawa and ECC cells transfected with pcDNA3.1/OIP5-AS1 or pcDNA3.1 via colony formation assays. (C) Apoptosis evaluation of transfected cells via flow cytometry assay. (D-E) Assessment of migration and invasion of transfected cells through the implementation of transwell assays. (F) Estimate of associated proteins of EMT in transfected cells by western blot. **P < 0.01.

## Data Availability

Data were not shared.

## Abbreviations

EC: Endometrial carcinoma
lncRNAs: Long non-coding RNAs
EMT: Epithelial–mesenchymal transition
miRNAs: MicroRNAs
URT: Untranslated region
RISCs: RNA-induced silencing complexes
ceRNA: Competing endogenous RNA
FBS: Fetal bovine serum
RT-qPCR: RNA extraction and real-time quantitative PCR
PBS: Phosphate-buffered saline
WT: Wild-type
Mut: Mutated
RIP: RNA immunoprecipitation
