# Supplementary figures and images for: OIP5-AS1 contributes to the development in endometrial carcinoma cells by targeting miR-152-3p to up-regulate SLC7A5
Source: Cancer Cell Int. 2021 Aug 21;21:440. doi: 10.1186/s12935-021-02061-0 (PMC8379738; doi:10.1186/s12935-021-02061-0)

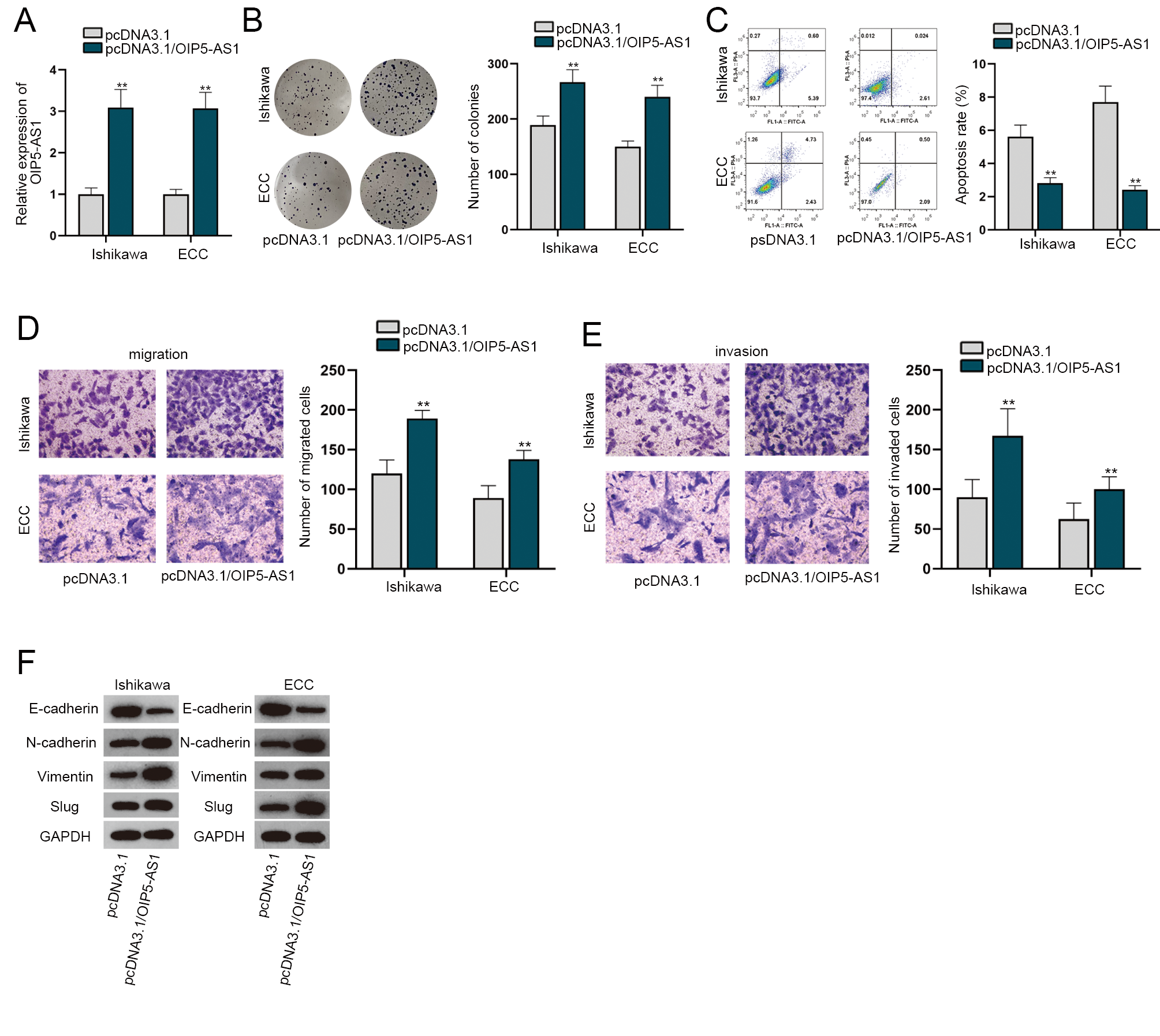

Supplement: Supplementary file 1 — Additional file 1: Figure S1. (A) Efficacy assessment of OIP5-AS1 overexpression via RT-qPCR in Ishikawa and ECC cells. (B) Detection of proliferation capacity of Ishikawa and ECC cells transfected with pcDNA3.1/OIP5-AS1 or pcDNA3.1 via colony formation assays. (C) Apoptosis evaluation of transfected cells via flow cytometry assay. (D-E) Assessment of migration and invasion of transfected cells through the implementation of transwell assays. (F) Estimate of associated proteins of EMT in transfected cells by western blot. **P < 0.01. [file 12935_2021_2061_MOESM1_ESM.tif]
